# World Allergy Organization (WAO) Diagnosis and Rationale for Action against Cow's Milk Allergy (DRACMA) Guidelines update – I – Plan and definitions

**DOI:** 10.1016/j.waojou.2021.100609

**Published:** 2022-02-01

**Authors:** Alessandro Fiocchi, Antonio Bognanni, Jan Brożek, Motohiro Ebisawa, Holger Schünemann, the WAO DRACMA guideline group, Ignacio J. Ansotegui, Stefania Arasi, Amal H. Assa'ad, Sami L. Bahna, Roberto Berni Canani, Martin Bozzola, Derek Chu, Lamia Dahdah, Christophe Dupont, Ramon Targino Firmino, Elena Galli, Rose Kamenwa, Gideon Lack, Haiqi Li, Alberto Martelli, Anna Nowak-Węgrzyn, Nikolas G. Papadopoulos, Ruby Pawankar, Maria Said, Mario Sánchez-Borges, Raanan Shamir, Jonathan M. Spergel, Hania Szajewska, Luigi Terracciano, Yvan Vandenplas, Carina Venter, Amena Warner, Susan Waserman, Gary W.K. Wong

**Affiliations:** f Department of Allergy & Immunology, Hospital Quironsalud Bizkaia, Erandio, Bilbao, Spain; g Translational Research in Pediatric Specialities Area, Division of Allergy, Bambino Gesù Children's Hospital, IRCCS, Erandio, Rome, Italy; h Division of Allergy and Immunology, Cincinnati Children's Hospital Medical Center, Cincinnati, OH, USA; i Allergy/Immunology Section, Louisiana State University Health Sciences Center, Shreveport, LA, USA; j Department of Translational Medical Science, University of Naples Federico II, Naples, Italy; k Department of Pediatrics, Pediatric Allergy/Immunology Section, British Hospital, Buenos Aires, Argentina; l Paris Descartes University, Pediatric Gastroenterology, Necker Hospital, Paris, Clinique Marcel Sembat, Boulogne-Billancourt, France; m Department of Medicine, Division of Clinical Immunology and Allergy, Department of Clinical Epidemiology & Biostatistics, McMaster University Health Sciences Centre, Erandio, Hamilton, ON, Canada; n Faculty of Medical Sciences of Campina Grande, UNIFACISA University Centre, Campina Grande, Paraiba, Brazil; o Pediatric Allergy Unit, Research Center, San Pietro-Fatebenefratelli Hospital, Rome, Italy; p Department of Pediatrics and Child Health, Aga Khan University Hospital, Nairobi, Kenya; q Department of Women and Children's Health/Peter Gorer Department of Immunobiology, School of Life Course Sciences, Faculty of Life Sciences & Medicine, King's College London, UK; r Evelina London Children's Hospital, Guy's and St Thomas' Hospital NHS Foundation Trust, London, UK; s Pediatric Division Department of Primary Child Care, Children's Hospital, Chongqing Medical University, Chongqing, China; t Italian Society of Pediatric Allergy and Immunology, Milano, Italy; u Department of Pediatrics, New York University Langone Health, New York, NY, USA; v Department of Pediatrics, Gastroenterology and Nutrition, Collegium Medicum, University of Warmia and Mazury, Olsztyn, Poland; w Allergy Unit, 2nd Pediatric Clinic, University of Athens, Athens, Greece; x Division of Infection, Immunity & Respiratory Medicine, University of Manchester, UK; y Department of Pediatrics, Nippon Medical School, Bunkyo-Ku, Tokyo, Japan; z Allergy & Anaphylaxis Australia (A&AA), Castle Hills, New South Wales, Australia; aa Department of Allergy and Clinical Immunology, Centro Médico-Docente La Trinidad Caracas, Venezuela; ab Institute of Gastroenterology, Nutrition and Liver Disease, Schneider Children's Medical Center, Petach-Tikva, Israel; ac Sackler Faculty of Medicine, Tel-Aviv University, Tel-Aviv, Israel; ad Division of Allergy and Immunology, Department of Pediatrics, The Children's Hospital of Philadelphia, Perelman School of Medicine at University of Pennsylvania, Philadelphia, PA, USA; ae The Medical University of Warsaw - Department of Paediatrics, Warsaw, Poland; af Italian NHS and Italian Society of Social and Preventive Pediatrics, Milano, Italy; ag Department of Pediatrics, UZ Brussel, Vrije Universiteit Brussel, Brussels, Belgium; ah Section of Allergy & Immunology, University of Colorado Denver School of Medicine, Children's Hospital Colorado, Aurora, CO, USA; ai Allergy UK, Planwell House, Sidcup, Kent, UK; aj Division of Clinical Immunology and Allergy, Department of Medicine, McMaster University, Hamilton, ON, Canada; ak Department of Paediatrics, Faculty of Medicine, The Chinese University of Hong Kong, Hong Kong, China; a Translational Research in Pediatric Specialities Area, Division of Allergy, Bambino Gesù Children's Hospital, IRCCS, Rome, Italy; b Department of Health Research Methods, Evidence and Impact (HEI), McMaster University, Hamilton, ON, Canada; c Department of Medicine, Division of Clinical Immunology and Allergy, Department of Clinical Epidemiology & Biostatistics, McMaster University Health Sciences Centre, Hamilton, ON, Canada; d Clinical Research Center for Allergy and Rheumatology, National Hospital Organization Sagamihara National Hospital, Kanagawa, Japan; e Cochrane Canada and McMaster GRADE Centre, Hamilton, Ontario, Canada

**Keywords:** Food allergy, Cow's milk allergy, Oral immunotherapy, GRADE

## Abstract

Since the World Allergy Organization (WAO) Diagnosis and Rationale against Cow's Milk Allergy (DRACMA) Guidelines were published 10 years ago, new evidence has accumulated about the diagnosis, therapy, and specific immunotherapy for cow's milk allergy (CMA). For this reason, WAO has felt the need to update the guidelines.

We introduce here this update. The new DRACMA guidelines aim to comprehensively address the guidance on diagnosis and therapy of both IgE non-IgE-mediated forms of cow's milk allergy in children and adults. They will be divided into 18 chapters, each of which will be dedicated to an aspect. The focus will be on the meta-analyzes and recommendations that will be expressed for the 3 most relevant clinical aspects: (a) the diagnostic identification of the condition; (b) the choice of the replacement formula in case of CMA in infancy when the mother is not able to breastfeed, and (c) the use of specific immunotherapy for cow's milk protein allergy.

## Introduction

IgE-mediated cow's milk protein allergy (IgE-CMA) has been a primary topic of interest for WAO since 2010, the year in which the first Grading of Recommendations Assessment, Development, and Evaluation (GRADE)-based guidelines on the management of this condition were published.^1^ Of notice, the World Allergy Organization (WAO) Diagnosis and Rationale against Cow's Milk Allergy (DRACMA) Guidelines had a noticeable impact on clinical practice regarding IgE-CMA, raising awareness on several aspects.^2^

First, the DRACMA guidelines presented a more nuanced and comprehensive diagnostic process, which, despite being generally based on oral food challenges (OFCs), could be supplemented, and in some cases replaced by an appropriate use of other tests such as skin prick test (SPT) and specific IgE determination (sIgE). The decision of which approach should be employed required an high-degree of personal contextualization, both depending on the specific circumstances and the values and preferences of the clinicians/patients.

Second, the guidelines pointed out the necessity by infants aged <2 years of a substitutive formula whenever their mother could not breastfeed, with the best choice being frequently cow's milk extensively Hydrolyzed Formula (eHF). Where available, Hydrolyzed Rice Formula (HRF) was considered equivalent, while Amino Acid Formulae (AAF) was to be reserved for the most severe cases. Soy formulae were generally deemed not to be a first choice, while milk from other mammals (eg, donkey, camel, mare, sheep, and ewe) was not to be used given the mismatch with the infants’ nutritional needs. Also in this case, the choice should rely on the context, and the values and preferences of the clinicians/patients.

Third, Oral Immunotherapy (OIT) with milk was considered as an experimental procedure, not suitable for routine clinical practice.^3^

Ten years later, despite the DRACMA methods still being valid,^4^ the scenario has dramatically evolved, prompting an update in guidance. Differently from other food allergies, reported by many as increasingly prevalent, CMA appears to have not undergone this trajectory.^5^^,^^6^ Even so, milk allergy remains a priority concern for allergists and pediatrician worldwide, with dairy anaphylaxis being now more common than peanut anaphylaxis, and the most frequently associated to lethal allergic reactions, as shown in a recent review on school-aged children with CMA.^7^

**Figure:**
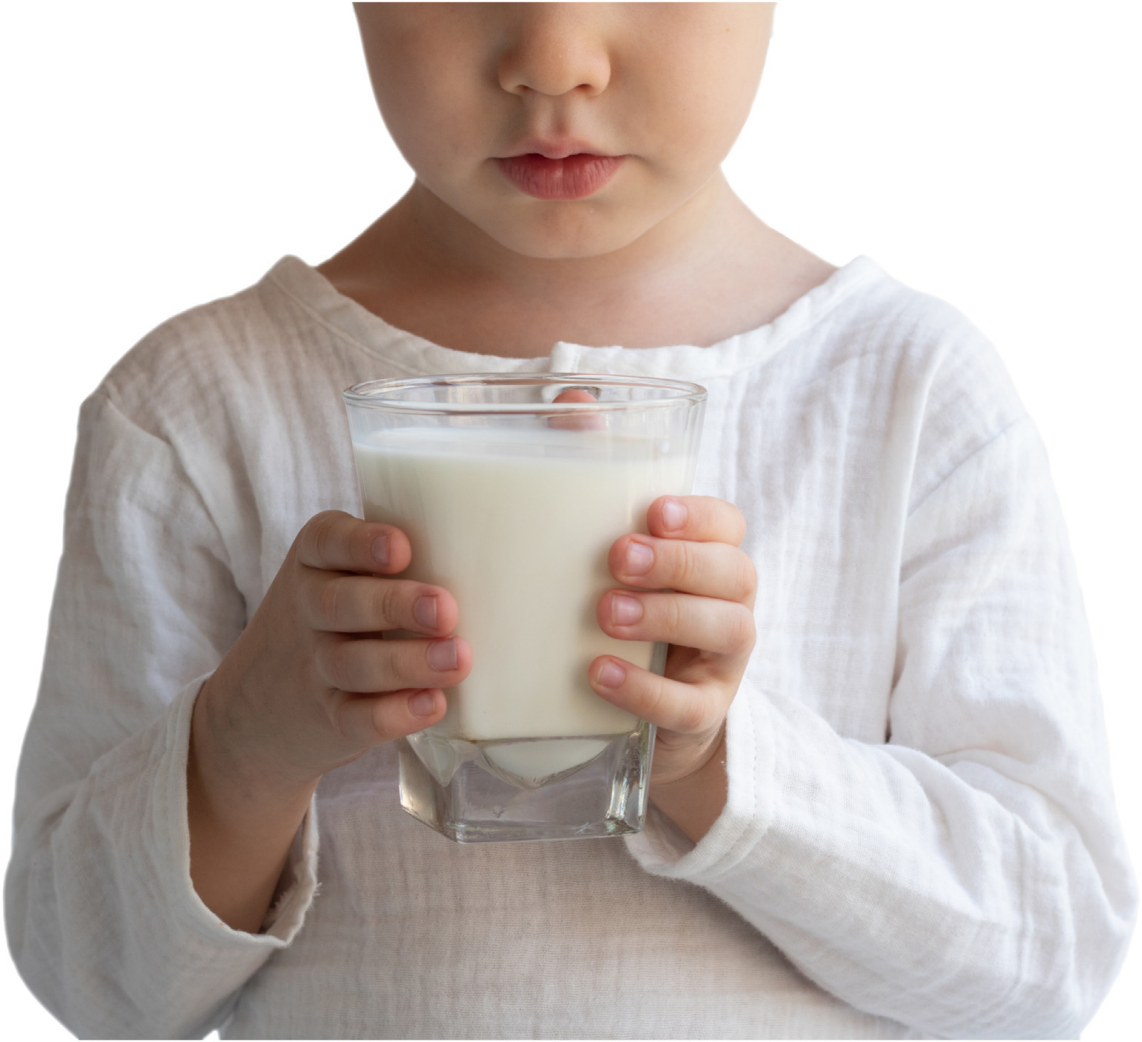

We introduce herein the updated DRACMA guidelines. We aim to illustrate the progress in diagnosis, therapy, and immunotherapy of IgE-CMA that could tailor the management of CMA. We will shortly indicate the guidelines published after DRACMA, over the decade 2010/2020. Finally, we will present the structure of the reviewed guidelines that took place between 2016 and 2021 and whose publication begins with this issue of the World Allergy Organization Journal. The new DRACMA guidelines aim to comprehensively address the guidance on diagnosis and therapy of both IgE non-IgE-mediated forms of CMA.

## 2010–2020: Open questions in diagnosis, therapy, and immunotherapy of CMA

The **diagnosis** was preached by the DRACMA guidelines on the use of the OFC as a "gold standard" for **IgE-CMA**. This somewhat bombastic definition emphasize the need of a scientifically correct diagnosis, in order to prevent CMA overdiagnosis. The OFC certainly retains its validity, but over the years, its limitations have become increasingly evident. For example, OFC results are not predictive of the severity of subsequent reactions.^8^ Also, there is no direct correlation between the eliciting threshold experienced by children during an OFC and the reaction's severity upon accidental exposure.^9^ Tools such as the Basophil Activation Test (BAT) have been developed to minimize the risk of severe reactions to the OFC,^10^^,^^11^ being also proposed as replacement tests of the OFC.^12^ In addition, serious reactions to the OFC have been described, up to a case of fatal reaction.^13^ These considerations will affect the direction of recommendations formulated by the guideline panel for the diagnosis of IgE mediated allergy. Other challenges inherent the diagnosis of IgE-CMA are the reassessment of the role of total and specific IgE assay, the interpretation of skin tests, and the possible role of molecular testing in diagnostic evaluation.^14^^,^^15^

Finally, as about 70% of IgE-CMA patients are found to tolerate baked milk, the latter might be considered for a role in the CMA diagnostic pathway, prior to fresh milk testing^16^

The elimination diet for milk, which prepares the OFC in IgE-mediated food allergy, completely replaces it in most guidelines for the diagnosis of **non IgE-mediated CMA** (non-IgE-CMA).^17^ We will see later how this might have profound influence over the epidemiologic estimates of the disease, which will be among the priority topics to be addressed in the new DRACMA guidelines. Specifically, we will try to address in an evidence-based manner the following questions: Should an elimination diet be followed by OFC in the individuals suspected of non-IgE-mediated CMA? Is there any use of atopy patch test to milk in these children? Is there any role for endoscopy ± biopsy in children with suspected milk-induced Eosinophilic Esophagitis (EoE) or non-esophageal Eosinophilic Gastrointestinal Disorders (EGIDs), including eosinophilic gastroenteritis and colitis? Are the diagnostic challenge procedures, recommended by specific guidelines for Food-Protein-Induced Enterocolitis Syndrome (FPIES),^18^ adequately informed by evidence?

**Figure:**
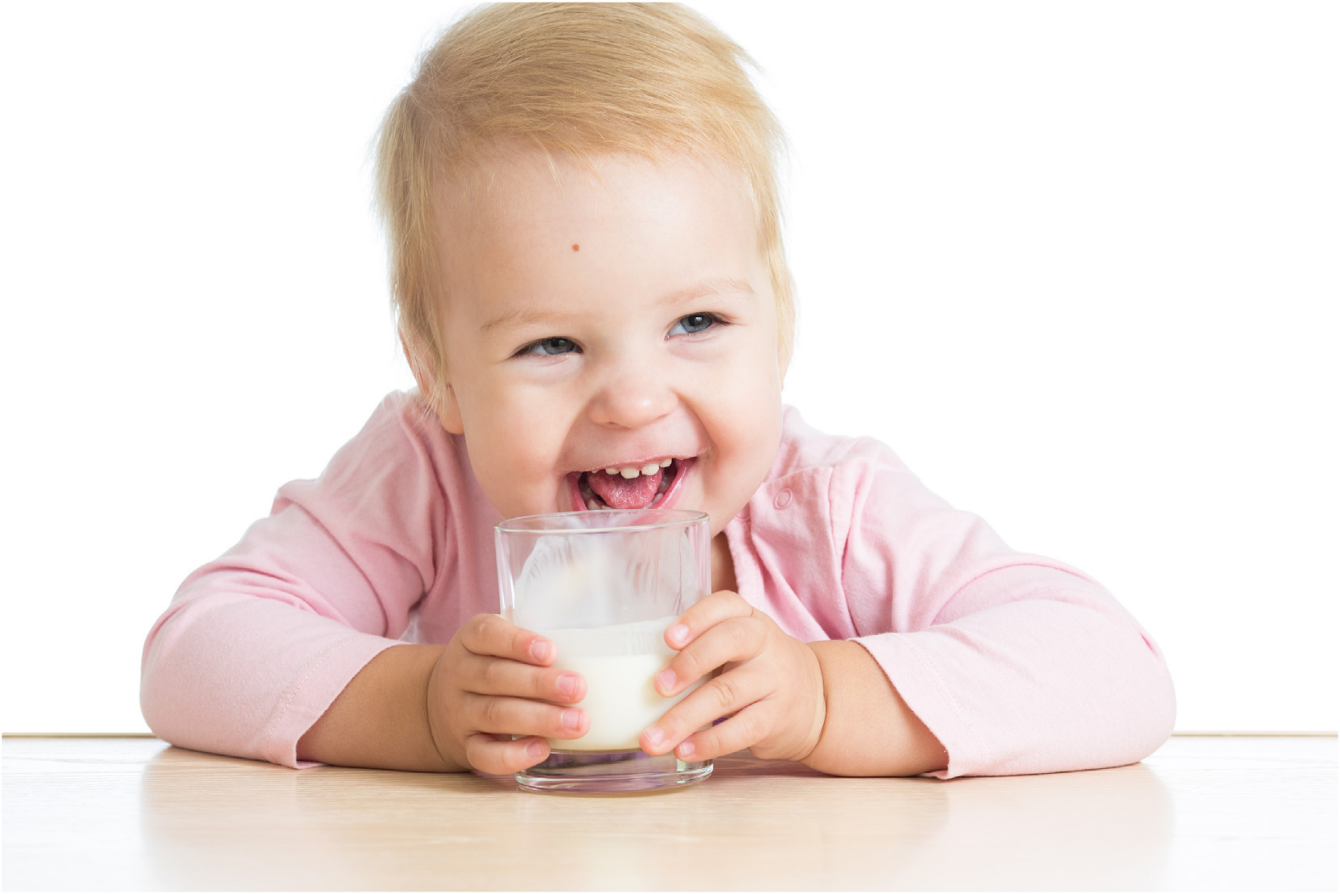

In synthesis, reconciling the diagnostic procedures for the different forms of CMA will be a challenge for the new DRACMA guidelines.^19^

A peculiar issue to consider, in the treatment of CMA, given the pivotal importance of maternal milk for children up to 24/36 months of age, is to confirm the evidence underlying the suggestion of cow's milk (CM) elimination diet for mothers breastfeeding allergic infants.^20^ In the past 10 years, the involvement of formulas in the management of CMA has been profoundly expanded, with extensively hydrolyzed formulas (eHFs),^21^ rice hydrolyzed formula (HRF),^22^ amino acid formulae (AAF),^23^ camel and dromedary milk,^24^ and donkey milk^25^ receiving increasing attention from the health community and being implemented in medical practice. To properly represent the change of the topic, we will update the systematic review investigating the effect of formulas in the management of CMA.

Another important aspect to account for is the reported effect of associating probiotics with formulas, either administered separately or mixed in the same formulation, on the duration of IgE-CMA.^26^^,^^27^ Another issue that will be investigated is the employment of new synbiotic-supplemented amino acid-based formulas.^28^^,^^29^

Over the course of the last decade, several advances have been done in developing novel protocols of CM oral immunotherapy, with the most notable examples being the weekly^30^ or slow up-dosing regimens,^31^ the rapid oral desensitization combined with omalizumab,^32^ different maintenance feeding regimens,^33^ and baked milk oral immunotherapy^34^, ^35^, ^36^

Previous systematic reviews investigating this aspect of IgE-CMA management were published in 2012 and 2017 including, but not limited to, OIT for IgE-CMA.^37^^,^^38^ The systematic review and guideline publication focusing on this topic will be the first among the 2021 DRACMA-related publications.

## CMA guidelines published after DRACMA

Since the first edition of DRACMA, other guidelines, consensuses, and position papers have been issued on CMA at the regional or national level. Some of them were national guidance items, implementing locally the DRACMA guidelines, others were *de novo* publications, developed using different methodologies. We report in Table 1 a list of the main CMA guidelines published over the course of the past 10 years.

**Table 1:** Main consensus, position papers, and guidelines produced worldwide between 2010 and 2020

Country/region	Issuing scientific society	Guideline identification	DRACMA based?	Main characteristics	Ref.
Europe	ESPGHAN	ESPGHAN CMPA guidelines	No	Focus on non-IgE CMA	^39^
Europe	European Academy of Allergy and Clinical Immunology (EAACI)	EAACI food allergy guidelines	No	Not limited to CMA	^40^
France	Société Française de Pédiatrie	Dietetic treatment of cow's milk protein allergy.	No	Limited to treatment	^41^
Italy	Emilian Working Group on Pediatric Allergy and Gastroenterology	A practical guide	No	Focus on diagnosis and management in primary care	^42^
Italy	Italian Society of Pediatric Allergy	DRACMA	Yes	Italian translation	^43^
United Kingdom	National Institute for Health and Care Excellence (NICE)	MAP (Milk Allergy in Primary Care)	No	Focus on non IgE-CMA in primary care	^44^
United Kingdom	NICE-derived	i-MAP (international MAP)	Partly	Focus on non IgE CMA in primary care	^45^
United Kingdom	British Society for Allergy and Clinical Immunology (BSACI)	BSACI cow's milk allergy guideline	No	Comprehensive	^46^
United Kingdom	NICE-derived	Updated i-MAP (international MAP)	Partly	Focus on CMA in primary care	^47^
Finland	Finnish Allergy Programme 2008–2018	Practical recommendations of the Finnish Allergy Programme 2008–2018 for prevention, diagnosis, and treatment	No	CMA as part of food allergy management in children	^48^
Spain	Spanish Society of Pediatric Clinical Immunology and Allergology (SEICAP)	Spanish CM guideline	Partly	Comprehensive	^49^
Spain	Spanish Society of Paediatric Gastroenterology, Hepatology, and Nutrition (SEGHNP), Spanish Association of Paediatric Primary Care (AEPAP), Spanish Society of Extra-hospital Paediatrics and Primary Health Care (SEPEAP), and the Spanish Society of Paediatric ClinicaL Immunology, Allergy, and Asthma (SEICAP)	Spanish CM guideline for non IgE-mediated CMA	No	Focus on non IgE CMA	^50^
Turkey	Turkish Society of Pediatrics	Turkish Consensus	Partly	Focus on primary care	^51^
Middle East	Independent group	Middle East consensus	Yes	Focus on primary care	^52^
India	Indian Society of Pediatric Gastroenterology, Hepatology and Nutrition	Indian Consensus	No	Focus on primary care	^17^
China	World Allergy Organization (WAO)	DRACMA	Yes	Mandarin translation	^53^
China	Independent group	Intensive DRACMA reading	Yes	Implementation in China	^54^
Japan	Japanese Society of Pediatric Allergy and Clinical Immunology (JSPACI); Japanese Society of Allergology (JSA)	Japanese guidelines	Partly	Not limited to CMA	^55^
Mexico	Independent group	GL-APLV	Partly	Comprehensive	^56^
South America	World Allergy Organization (WAO)	DRACMA	Yes	Spanish translation	^57^

Among the publications above, the one most implemented is the UK NICE – derived guideline, the iMAP guideline. It includes an algorithm for the diagnostic and therapeutic approaches, based on the heterogeneous clinical manifestations of CMA (both non-IgE and IgE).^45^ Interestingly, the diagnostic process for CMA accounts for a diagnosis not confirmed through OFC, given that a series of conditions are met (improvement on a strict cow's milk protein-free elimination diet for at least 2 weeks; clinical relapse on subsequent cow's milk open challenge), possibly leading to an overestimation of non-IgE-CMA. After their implementation in Northern Ireland, the use of hypoallergenic formulas largely increased, exceeding the expected epidemiological figures^58^^,^^59^

The quality of guidelines on CMA, published between 2010 and 2015, was assessed through the Appraisal of Guidelines for Research and Evaluation (AGREE II) tool.^60^ The appraisal highlighted the lack of a defined quality standard, as only 3 presented satisfactory scores across the key domains. In light of this, in the present update of the DRACMA guidelines we strive to adhere to the highest methodological standards in the evaluation of evidence and its translation into recommendations.

## Methods applied in the 2021 DRACMA guidelines

We followed the Grading of Recommendations Assessment, Development, and Evaluation (GRADE) approach {PMID: 21195583} and the European Commission methods for developing practice guidelines.^61^ WAO established a multidisciplinary guideline panel (DRACMA Scientific Committee) composed of content experts and representatives of key stakeholders, including patient representatives, nutritionists, and general practitioners. All panel members declared their actual, potential, and/or perceived competing interests. Those were reviewed by an anonymous WAO committee that decided which panel members should abstain from voting on selected recommendations related to immunotherapy, formulas, and diagnosis of CMA.

A group of methodology experts from the McMaster GRADE Centre performed systematic reviews of the evidence and led the process of developing recommendations.

The DRACMA guideline panel generated a set of 61 questions and determined their priority to be answered with recommendations (Supplementary material). The methodology group performed necessary systematic reviews and prepared GRADE summary of findings tables. The voting panel members followed the evidence-to-decision (EtD) framework to develop recommendations either by in-person or online discussion following the modified Delphi approach. We published all decisions and the rationale for the recommendations as appendices to the guidelines.

## General structure of the 2021 DRACMA guidelines

The original guideline comprised 19 chapters merged into a single publication. This time we decided to publish the chapters separately in a dedicated series in the World Allergy Organization Journal to facilitate the dissemination and the implementation of the guideline. For this reason the chapters have been separated, and every topic will be published in a single article.

Table 2 shows the publication plan. Due to peer review process, the articles will not necessarily be published in the order indicated. We will start with the guidelines on OIT, those for which a greater harvest of new data has been produced. The guideline is submitted together with the metanalysis supporting it. Other articles will be published regularly, so that the project will configure a *Summa* of the relevant information about CMA*.*

**Table 2:** Plan of the DRACMA publications

	Topic	Method of preparation
General		
1.	Overview and definitions	This paper
2.	CMA epidemiology and natural history	Narrative review
3.	CM allergens and immunologic mechanisms	Narrative review
4.	Clinical presentations: IgE-mediated	Narrative review
5.	Clinical presentations: non IgE-mediated	Narrative review
6.	Comparison among different guidelines	Systematic review
7.	DRACMA methodology	Synthesis of methods
CMA diagnosis		
8.	Diagnosis of CMA	Systematic review
9.	Recommendations on CMA diagnosis	Guideline
Treatment options		
10.	Breastfeeding a baby with CMA	Narrative review
11.	Substitutive formulae	Systematic review
12.	Recommendations on substitutive treatment	Guideline
13.	Oral Immunotherapy for CMA	Systematic review
14.	Recommendations on CMA OIT	Guideline
15.	Other milks (goat's, ewe's, mare's, donkey's, camel's, and substitutes from non-animal sources)	Narrative review
16.	Nutritional considerations in CMA infants	Narrative review
Conclusions		
17.	Which is the 1st choice formula case by case?	Synthesis of recommendations
18.	Unmet needs. Recommendations for research. Recommendation for the implementation of the DRACMA guidelines. Periodical update of DRACMA.	Synthesis of recommendations

## Glossary of CMA

In developing the metanalyses and the guidelines, we adhered to the following definitions:

- **Cow's milk hypersensitivity** indicates non-allergic hypersensitivity (traditionally termed “cow's milk intolerance”) and allergic milk hypersensitivity
- **Cow's milk allergy** (CMA) indicates *“a hypersensitivity reaction initiated by specific immunological mechanisms*”^62^
- **IgE-mediated CMA** (IgE-CMA) indicates a hypersensitivity reaction to cow's milk proteins initiated by specific Immunoglobulin E binding to Fcε receptors on effector cells as mast cells and basophils. This causes release of histamine and other preformed mediators, and rapid symptom onset.^63^
- **Non IgE-mediated CMA** (non-IgE-CMA) indicates a hypersensitivity reaction to cow's milk proteins initiated by non-IgE mediated (mainly cell-mediated) mechanisms. Non-IgE-mediated milk reactions are typically delayed in onset
- **Anaphylaxis** is defined according to the amended WAO criteria for the diagnosis of anaphylaxis.^64^

Many other definitions of clinical presentations and pathologic mechanisms have been adopted during the development of the guidelines. When necessary, they will be specified in the respective papers.

## What is next

One of the determinants of the profound heterogeneity in the management of CMA consists in the wide spectrum of professional figures (paediatrician, allergists, gastroenterologists, and so forth) dealing with it. Another is the contradictory guidance provided by a large number of guidelines and position papers. As a consequence, the 2021 updated DRACMA guidelines aim to comprehensively address the diagnostic and therapeutic fields of CMA, harmonizing the collaboration between the various specialist figures.

By their very nature, guidelines make clarity. Clarity is bound to reduce both underdiagnosis and especially overdiagnosis of CMA. We hope we have done the allergy community a good service, and we apologize right now if something went wrong.

## Mario Sánchez-Borges

Before proceeding with the publication of the guidelines, we want to celebrate the remarkable life and academic accomplishments of one of our fellow authors. Mario Sánchez-Borges, MD, was a true leader for the entire international allergy community, without whose guidance and contribution, the realization of these guidelines would not have been possible. As a previous WAO president (2016–2017) and Councilor, Mario has been an impulse and prime mover of DRACMA. He participated in the drafting of all the parts that will report him as author. His kindness and generosity will stay unperished, living through the numerous and joyful memories he left in so many of us.

**Figure:**
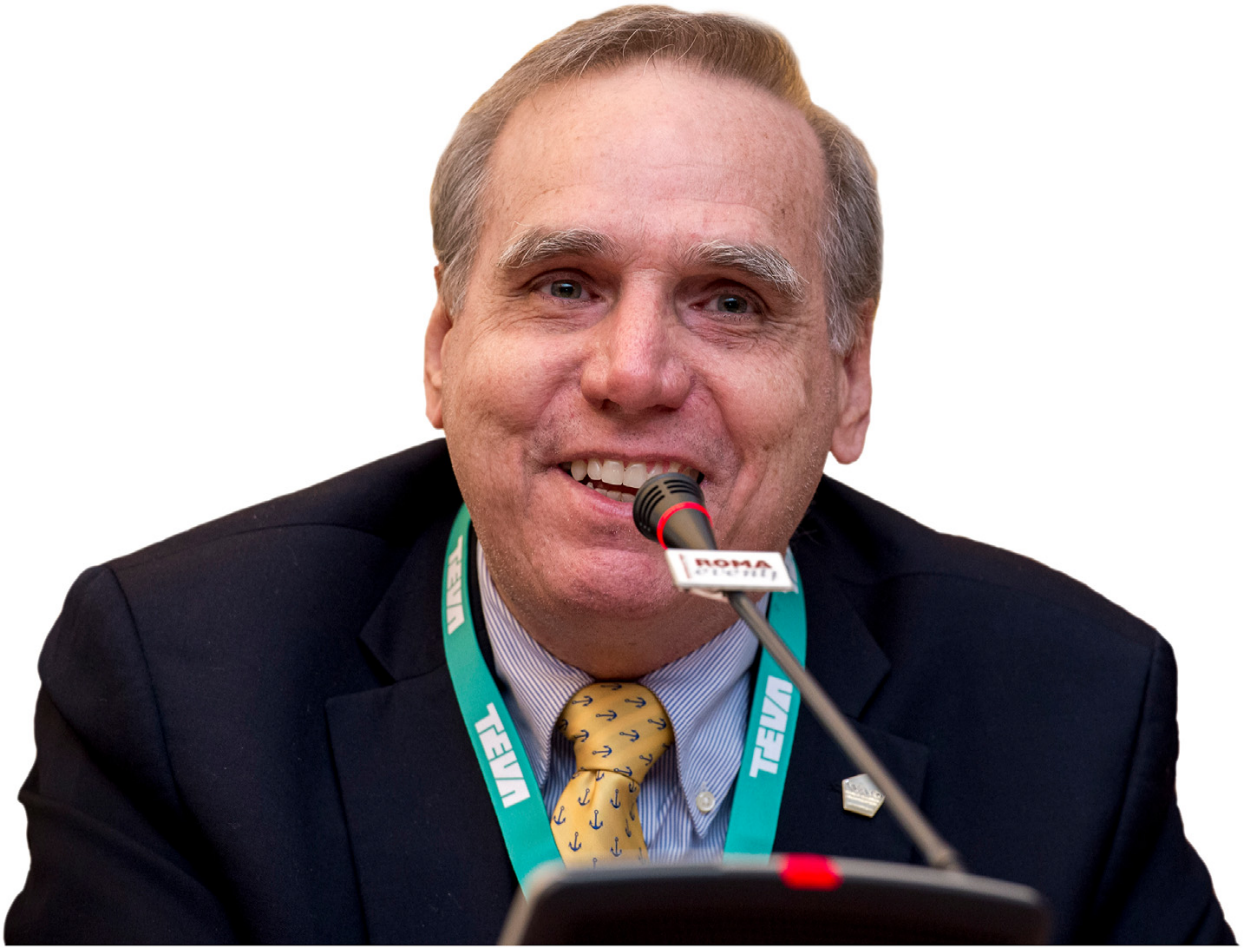

## Consent to publish

All authors agree to the publication of this manuscript in World Allergy Organization Journal.

## Ethics statement

This manuscript is an editorial. It did not involve human subjects.

## Author contributions

AF initiated the concept and contributed made the first draft. AB, JB, ME, and HS participated in the development of the document. All authors reviewed and approved the final manuscript.

## Funding

This document was supported by the World Allergy Organization.

## Declaration of competing interest

S Arasi, S Bahna, Bognanni, J Brożek, D Chu, L Dahdah, E Galli, R Kamenwa, H Li, A Martelli, R Pawankar, H Schünemann, R Targino, L Terracciano, and A Warner have no conflicts to disclose. Relationships reported related to the submitted work: IJ Anstotegui – Abbott, Amgen, Astra Zeneca, Bayer, Bial, Faes Farma, Hikma, Menarini, Merck, Mundipharma, Roxall, Sanofi, Stallergenes, UCB. A Assa’ad – Aimmune Therapeutics, DBV Technologies, Astella, ABBVIE, Novartis, Sanofi, FARE, NIH and an intellectual property patent licensed to Hoth. R Berni Canani – Ch.Hansen, Danone, DVB, Humana, iHealth, Kraft Heinz, Mead Johnson, Nestlè, Novalac, Nutricia, Sanofi. M Bozzola – Danone C Dupont – Nestle Health Science, Nestle France, Nutricia, Novalac, Sodilac, Abbott, Danone, and stock ownership at DBV Technologies. M Ebisawa – DBV Technologies, Mylan, ARS Pharmaceuticals, Novartis. A Fiocchi – Abbott, Danone. G Lack – FARE, National Peanut Board (NPB), The Davis Foundation, Action Medical Research, UK Food Standards Agency, Medical Research Council, DBV Technologies, Mission Mighty Me, Novartis, Sanofi-Genyzme, Regeneron, ALK-Abello, Lurie Children's Hospital. A Nowak-Wegrzyn – Nestle, Nutricia, Novartis, Gerber, Aimmune. N Papadopoulos – Novartis, Nutricia, HAL Allergy, Menarini/Faes Farma, Sanofi, Mylan/Meda, Biomay, AstraZeneca, GSK, MSD, ASIT Biotech, Boehringer Ingelheim, Gerolymatos International SA, Capricare. M Said – Nestle, Nutricia, Abbott, Bayer for Anaphylaxis Australia. J Spergel – DBV Technologies, Regeneron, Sanofi, and Aimmune. H Szajewska – Ausnutria, Cargill, Danone, Else Nutrition, Hipp, Nestle, and Nestle Nutrition Institute. Y Vandenplas – Abbott Nutrition, Biogaia, Biocodex, By Heart, CHR Hansen, Danone, ELSE Nutrition, Friesland Campina, Hero, Hypocrata, Nestle Health Science, Nestle Nutrition Institute, Nutricia, Mead Johnson Nutrition, Orafti, Phacobel, Phathom Pharmaceuticals, Sari Husada, United Pharmaceuticals (Novalac), Wyeth, Yakult. C Venter – Reckitt Benckiser, Nestle Nutrition Institute, Danone, Abbott Nutrition, Else Nutrition, and Before Brands, DBV Technologies. S Waserman – Novartis-basic science work on peanut allergy, Aimmune-peanut OIT trial, Medical Advisor to Food Allergy Canada, and Pfizer, Bausch, Kaleo-consultant for epinephrine autoinjectors. GWK Wong – Nestle, Danone.
