# Supplementary material for: World Allergy Organization (WAO) Diagnosis and Rationale for Action against Cow's Milk Allergy (DRACMA) Guidelines update – I – Plan and definitions
Source: World Allergy Organ J. 2022 Feb 1;15(1):100609. doi: 10.1016/j.waojou.2021.100609 (PMC8818560; doi:10.1016/j.waojou.2021.100609)
Supplement: Multimedia component 1 [file mmc1.docx]

**Table 2 – questions generated by a survey among panel members, and their ratings.**

**1. Questions that we will attempt to answer in this update of DRACMA**

**TREATMENT OF CMA**

| **1.** | **Formulas** | **Priority** |
| --- | --- | --- |
| 1.1. | eHF vs. amino acid formula for **IgE-mediated CMA** | 1 |
| 1.1.N | eHF vs. amino acid formula for **non-IgE-mediated CMA** | 1 |
| 1.2. | eHF vs. extensively hydrolyzed rice formula for **IgE-mediated CMA** | 4 |
| 1.2.N | eHF vs. extensively hydrolyzed rice for **non-IgE-mediated CMA** | 3 |
| 1.3. | eHF vs. soy formula for **IgE-mediated CMA** | 4 |
| 1.3.N | eHF vs. soy formula for **non-IgE-mediated CMA** | 3 |
| 1.4. | extensively hydrolyzed rice formula vs. soy formula for **IgE-mediated CMA** | 4 |
| 1.4.N | extensively hydrolyzed rice formula vs. soy formula for **non- IgE-mediated CMA** | 4 |
| **2.** | **OIT** |  |
| 2.1. | OIT with milk vs. strict avoidance of milk alone for children and adults with **IgE-mediated CMA** | 1 |
| 2.2. | OIT with baked milk vs. strict avoidance of milk alone for **IgE-mediated CMA** | 1 |
| 2.3. | OIT with baked milk vs. OIT with fresh milk for **IgE-mediated CMA** | 1 |
| 2.4. | omalizumab + OIT vs. OIT alone for **IgE-mediated CMA** | 4 |
| **3.** | **EIT** |  |
| 3.1. | epicutaneous IT with milk vs. OIT with milk for IgE-mediated CMA | 3 |
| **4.** | **Diet for breastfeeding mothers** |  |
| 4.1. | diet without milk vs. no specific diet for breastfeeding mothers of infants with **IgE-mediated CMA** | 4 |
| 4.1.N | diet without milk vs. no specific diet for breastfeeding mothers of infants with **non-IgE-mediated CMA** | 3 |
| **5.** | **Small amounts of milk** |  |
| 5.1. | small amounts of milk vs. strict avoidance milk for patients who reacted to high doses of milk during OFC | 3 |
| **6.** | **Baked milk** |  |
| 6.1.N | extensively heated (baked) milk vs. strict avoidance of milk for **non-IgE-mediated CMA** | 4 |
| 6.2.N | extensively heated (baked) milk vs. formulas for **non-IgE-mediated CMA** | 4 |

**DIAGNOSIS OF CMA**

| **D1.** | **Oral food challenge** | **Priority** |
| --- | --- | --- |
| D1.1.N | elimination diet followed by OFC vs. elimination diet without OFC in those suspected of **non-IgE-mediated CMA** | 1 |
| **D2.** | **SPT & sIgE** |  |
| D2.1. | SPT with milk vs. OFC with milk in patients **suspected of IgE-mediated CMA** | 2 |
| D2.1.E | SPT with milk vs. OFC with milk in patients **suspected of** **CMA-induced EoE** | 3 |
| D2.2. | sIgE vs. OFC with milk in patients **suspected of IgE-mediated CMA** | 2 |
| D2.3. | sIgE to whole milk and milk components* vs. OFC with milk in patients **suspected of IgE-mediated CMA** | 3 |
| D2.4. | SPT with milk + sIgE vs. OFC with milk in patients **suspected of IgE-mediated CMA** | 3 |
| D2.5. | SPT with milk + sIgE to whole milk and milk components* vs. OFC with milk in patients **suspected of IgE-mediated CMA** | 3 |
| D2.6. | sIgE vs. SPT with milk in patients **suspected of IgE-mediated CMA** | 2 |
| **D3.** | **Testing for allergens other than milk** |  |
| D3.1. | testing (SPT, sIgE) for sensitization to milk + other food allergens (hen’s egg, soy, beef meat, rice) vs. testing with milk only in children **suspected of IgE-mediated CMA** | 3 |
| **D4.** | **Testing for sensitization to baked milk** |  |
| D4.1. | testing for sensitization/allergy to baked milk vs. no testing before inclusion of baked milk in diet in patients with **confirmed IgE-mediated CMA** | 4 |
| D4.2. | SPT with baked milk vs. OFC with baked milk for diagnosis of tolerance to baked milk in patients with **confirmed IgE-mediated CMA** | 3 |
| D4.3. | testing for sensitization to cow’s milk components* (component resolved diagnostics) with either SPT, specific IgE, or microarray vs. OFC with baked milk for diagnosis of tolerance to baked milk in patients with **confirmed IgE-mediated CMA** | 4 |
| **D5.** | **Patch test** |  |
| D5.1.N | atopy patch test to milk vs. elimination diet alone in children with **suspected of non-IgE-mediated CMA** | 4 |

**2. Questions that were deemed a less important and there was no agreement about them among panel members**

**TREATMENT OF CMA**

**7. Probiotics**

7.1. formula + probiotics vs. formula alone for **IgE-mediated CMA**

7.1.N formula + probiotics vs. formula alone for **non-IgE-mediated CMA**

**8. Epinephrine**

8.1. Should epinephrine (adrenaline) autoinjector vs. no autoinjector be prescribed for patients with **IgE-mediated CMA**?

**DIAGNOSIS OF CMA**

**D6. GI endoscopy**

D6.1.N upper GI endoscopy ±biopsy in children with **suspected milk-induced EoE**

D6.2.N lower GI endoscopy ±biopsy in children with **suspected non-IgE-mediated CMA**

**3. Questions that were judged less important and will not be answered in this update of DRACMA**

**TREATMENT OF IGE-MEDIATED CMA**

- avoidance of beef meat vs. no specific meat avoidance for IgE-mediated CMA
- camel milk vs. formula for infants with IgE-mediated CMA
- sheep milk vs. formula for infants with IgE-mediated CMA
- goat milk vs. formula for infants with IgE-mediated CMA
- donkey milk vs. formula for infants with IgE-mediated CMA
- omalizumab vs. avoidance diet alone for IgE-mediated CMA
- omalizumab vs. OIT with fresh milk for IgE-mediated CMA
- Should all patients with CMA be under the care of a dietitian vs. as needed?

**TREATMENT OF NON-IGE-MEDIATED CMA**

- camel milk vs. formula for non-IgE-mediated CMA
- sheep milk vs. formula for non-IgE-mediated CMA
- goat milk vs. formula for non-IgE-mediated CMA
- donkey milk vs. formula for non-IgE-mediated CMA
- Should epinephrine (adrenaline) autoinjector vs. no autoinjector be prescribed for patients with non-IgE-mediated CMA?

**DIAGNOSIS OF IGE-MEDIATED CMA**

- allergen microarray vs. OFC in patients suspected of IgE-mediated CMA
- basophil activation test with milk vs. OFC with milk in children suspected of IgE-mediated CMA
- sIgE to boiled milk vs. OFC with baked milk for diagnosis of tolerance to baked milk in patients with confirmed IgE-mediated CMA
- SPT with baked milk and with casein vs. OFC with baked milk for diagnosis of tolerance to baked milk in patients with confirmed IgE-mediated CMA
- sIgE to boiled milk and to casein vs. OFC with baked milk for diagnosis of tolerance to baked milk in patients with confirmed IgE-mediated CMA
- basophil activation test with baked milk vs. OFC with baked milk for diagnosis of tolerance to baked milk in patients with confirmed IgE-mediated CMA
- screening for sensitization to cow’s milk with SPT or sIgE to fresh milk vs. no screening in asymptomatic infants before the first introduction of cow's milk

**DIAGNOSIS OF NON-IGE-MEDIATED CMA**

- serum milk-specific IgG (and/or IgG subclasses) vs. OFC with milk in patients suspected of non-IgE-mediated CMA
- SPT with milk vs. OFC with milk in patients suspected of non-IgE-mediated CMA
- atopy patch test to milk vs. OFC with milk in patients suspected of cow’s milk-induced EoE, eczema, FPIES
- basophil activation test with milk vs. OFC with milk in patients suspected of non-IgE-mediated CMA
- T-cell activation test with milk vs. OFC with milk in patients suspected of non-IgE-mediated CMA
- fecal elastase in children with suspected milk-induced FPIES, enteropathy, or proctocolitis
- fecal leukocyte count in children with suspected milk-induced FPIES, enteropathy, or proctocolitis
- fecal calprotectin in children with suspected milk-induced FPIES, enteropathy, or proctocolitis
- fecal occult blood in children with suspected milk-induced FPIES, enteropathy, or proctocolitis
